# Impact of Particulate Matter 2.5 on Neurological Diseases: Insights Into Pathophysiological and Molecular Mechanisms

**DOI:** 10.1155/jt/5752904

**Published:** 2025-12-30

**Authors:** Carmen Rubio, Alejandro López-Landa, Norma Serrano-García, Héctor Romo-Parra, Moisés Rubio-Osornio

**Affiliations:** ^1^ Neurophysiology Department, National Institute of Neurology and Neurosurgery (MVS), Mexico City, Mexico; ^2^ Faculty of Medicine, Benemérita Autonomous University of Puebla, Puebla, Mexico; ^3^ Psychology Department, Ibero-American University, Mexico City, Mexico, ibero.mx; ^4^ Neurochemistry Department, National Institute of Neurology and Neurosurgery (MVS), Mexico City, Mexico

**Keywords:** neurodegenerative diseases, neuroinflammation, oxidative stress, PM2.5 exposure

## Abstract

**Background:**

Fine particulate matter (PM2.5) has been significantly linked to the progression of various neurological and neurodegenerative diseases.

**Objective:**

This review aims to elucidate the molecular and pathophysiological effects induced by chronic exposure to PM2.5 in neurological and neurodegenerative diseases, including Alzheimer’s, Parkinson’s, Huntington’s, multiple sclerosis, and epilepsy.

**Introduction:**

PM2.5 penetrates the central nervous system (CNS) via the olfactory nerve or by disrupting the blood–brain barrier (BBB), triggering oxidative stress, neuroinflammation, mitochondrial dysfunction, and epigenetic alterations.

**Discussion:**

In Alzheimer’s and Parkinson’s diseases, PM2.5 exacerbates the accumulation of β‐amyloid, hyperphosphorylated tau, and α‐synuclein, while in Huntington’s disease, it worsens toxicity mediated by mutant huntingtin. In multiple sclerosis, these particles intensify neuroinflammation and axonal damage, whereas in epilepsy, they promote neuronal hyperexcitability and recurrent seizures. These mechanisms contribute to neuronal damage, symptom progression, and functional decline.

**Conclusion:**

This evidence highlights the urgent need for strict environmental policies to reduce PM2.5 exposure and further research to develop therapeutic strategies that mitigate its effects on neurological diseases, thereby improving the health of vulnerable populations.

## 1. Introduction

**Figure 1:**
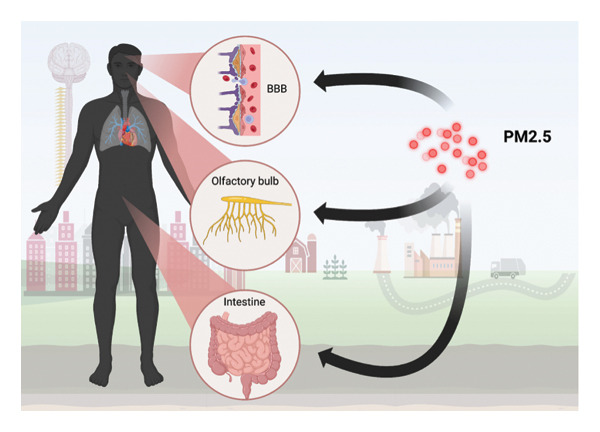
Pathways of PM2.5 access to the central nervous system. PM2.5 bypasses biological barriers blood–brain barrier [BBB], olfactory bulb, and gut) to penetrate the brain and other structures. Created with https://www.biorender.com/.

Fine particulate matter with a diameter less than 2.5 μm (PM2.5) poses a considerable threat in environmental pollution owing to its detrimental health consequences [1–4]. These ultrafine particles emanate from sources including fossil fuel combustion, industrial pollutants, agricultural practices, and natural events such as wildfires [5–8]. They comprise heavy metals including lead, mercury, arsenic, and nickel; minerals such as silica and aluminum [9, 10]; and both organic and inorganic constituents such as polycyclic aromatic hydrocarbons (PAHs), organic carbon, sulfates, and ammonium [11–16]. Owing to their diminutive dimensions, PM2.5 particles can remain suspended in the atmosphere for prolonged durations, enabling inhalation and subsequent infiltration into the pulmonary alveoli and systemic circulation [17–19]. Upon entering systemic circulation, these particles can traverse the BBB, undermining its integrity and infiltrating the CNS [20]. Alternative methods of entrance encompass direct anatomical contact through the olfactory nerve (Figure 1), and a recently hypothesized third route: the gut–brain axis, wherein particles modify microbiota composition following gastrointestinal exposure [21–23]. PM2.5 circumvents protective biological barriers, directly interacting with brain tissue [20, 24, 25], thereby instigating and exacerbating pathological processes within the nervous system [26–28], including stroke, neurodegenerative disorders such as Alzheimer’s disease (AD) and Parkinson’s disease (PD), multiple sclerosis (MS), and various neuropsychiatric conditions [29–33] (Table 1). Current research suggests that exposure to heightened PM2.5 concentrations leads to preliminary signs of neurodegenerative damage, including mitochondrial dysfunction, oxidative stress, microglial activation, vascular impairment, blood–brain barrier disruption, and lipid imbalance [55, 56]. Fundamental mechanisms such as neuroinflammation, epigenetic alterations, and disrupted cellular signaling are crucial for comprehending these impacts [57, 58]. PM2.5 contains many reactive chemicals and heavy metals that facilitate the generation of reactive oxygen species (ROS) and reactive nitrogen species, hence disrupting critical cellular processes, especially those related to mitochondrial function [59–61]. Subsequent oxidative damage to proteins and DNA leads to the buildup of pathogenic proteins, as evidenced in AD [30, 62]. Moreover, PM2.5 induces significant redox imbalance, resulting in alterations to molecular pathways that disrupt homeostasis and initiate epigenetic modifications, such as changes in DNA methylation and histone acetylation [1, 57, 63]. These alterations deregulate genes associated with synaptic plasticity, stress response, and cellular repair mechanisms [64]. Furthermore, PM2.5 induces neuroinflammation by stimulating immunological responses in both the peripheral and central neurological systems [48, 65]. Microglia, upon detecting PM2.5, secrete proinflammatory cytokines, including tumor necrosis factor‐alpha [TNF‐α] and interleukin‐6 [IL‐6], which exacerbate neuronal injury and disrupt homeostasis. These inflammatory processes facilitate the onset and progression of PD, wherein α‐synuclein aggregation impairs dopaminergic signaling, exacerbating both motor and nonmotor symptoms [66–68]. This study seeks to clarify the molecular and pathophysiological effects of chronic PM2.5 exposure on neurodegenerative illnesses, specifically AD, PD, MS, and epilepsy, due to the significant threat PM2.5 poses to neurological health. Elucidating these pathways may guide the formulation of preventive strategies and innovative therapy methods to alleviate neurotoxic harm linked to airborne pollution.

**Table 1:** Summary of molecular effects of PM2.5 exposure by neurological disease.

Neurological disease	Molecular targets and biomarkers	Key findings	PM2.5 concentration/exposure	Population/model	Reference
Alzheimer’s disease (AD)	Aβ1–40/42, BACE, PS1, GFAP, Iba1, CD68, COX‐1/2, IL‐1β, TNF‐α, IL‐6, IFN‐γ	Aβ accumulation, tau hyperphosphorylation, neuroinflammation, synaptic dysfunction, memory impairment	11–65 μg/m^3^; durations from 3 to 14 months	APP/PS1, TgF344‐AD, 3xTg‐AD mice, humans (MCMA)	[34–40]
Parkinson’s disease (PD)	α‐synuclein, TNF‐α, IL‐1β, IL‐6, JNK/ERK/p38 MAPK, AKT, NF‐κB, ROS	Upregulation of α‐synuclein and inflammatory markers, activation of MAPK and NF‐κB pathways, mitochondrial dysfunction	12–65 μg/m^3^; durations from 1 h to 10 months	C57BL/6 mice, HUVEC, BV‐2/HMC3 cells	[41–43]
Neurodevelopmental disorders	miRNAs (miR‐6315, miR‐3588, miR‐99b), BDNF, GAP43, PSD95, SYP	Altered neurogenesis, reduced synaptic proteins, impaired fetal learning and motor coordination	0.25–25 μg/mL; PND 3–15	Pregnant SD rats, offspring	[44, 45]
Epilepsy/neuroinflammation	NLRP3, ASC, IL‐1β, IL‐18, Caspase‐1, GSDMD	Activation of inflammasome and pyroptosis, enhanced pro‐inflammatory cytokines	25–50 μg/mL; 6 h	BV‐2, HMC‐3 microglia	[46]
Other CNS conditions/mixed	TNF‐α, IL‐1β, iNOS, ICAM‐1, COX‐2, NO, ROS, NF‐κB, AhR‐CYP1A1, Humanin, MOTS‐c	Vascular inflammation, mitochondrial dysfunction, oxidative stress, altered TCA cycle, synaptic damage	3–100 μg/mL; durations from 24 h to months	BV‐2 cells, HUVEC, C57BL/6 mice, ApoE−/− mice	[47–52]
Cognitive impairment/memory	BDNF, CREB, PKA, PSD95, GAP43, SYP	Decreased neuroplasticity markers, impaired learning/memory, hippocampal neurogenesis inhibition	12–595 μg/m^3^; durations from 28 days to > 10 years	Sprague‐Dawley rats, humans (MCMA)	[53, 54]
Neurovascular/endothelial dysfunction	eNOS, ROS, ICAM‐1, NO, iNOS, NF‐κB, TLR4	Endothelial dysfunction, inflammation, AhR pathway activation	4 mg/kg, 10 μg/day intranasal	ApoE−/−, C57BL/6, HUVEC	[49, 50, 52]

*Note:* Overview of the molecular and cellular impacts linked to PM2.5 exposure in several neurological disorders. The table delineates essential molecular targets and biomarkers, pertinent discoveries, PM2.5 concentrations and exposure durations, experimental models or human populations examined, and associated references.

## 2. Epilepsy

The correlation between PM2.5 and diseases like Alzheimer’s and Parkinson’s is widely documented, although its possible involvement in epilepsy is becoming increasingly recognized. Epilepsy is a neurological condition defined by atypical neuronal discharges that result in recurring seizures. PM2.5 may facilitate epileptogenesis through physiological, cellular, and molecular pathways that affect neuroinflammation, synaptic plasticity, and neuronal excitability [69, 70].

### 2.1. PM2.5 Infiltration and Blood–Brain Barrier Compromise

As previously discussed, PM2.5 can infiltrate the central nervous system via the olfactory nerve or by breaching the BBB [20]. Disruption of the BBB has been associated with increased neuronal hyperexcitability, a critical element in the onset of epilepsy [71]. Exposure to PM2.5 compromises BBB integrity by provoking oxidative and inflammatory reactions in endothelial cells, hence enabling immune cell infiltration into the brain parenchyma [51, 72, 73]. This increases the susceptibility of neural networks to epileptic activity.

### 2.2. Neuroinflammation and Microglial Activation

Microglial overactivation results in increased concentrations of IL‐1β, IL‐6, TNF‐α, and IFN‐γ, which disrupt neuronal signaling and diminish seizure thresholds [48, 74]. These proinflammatory cytokines disturb synaptic equilibrium and facilitate seizure propagation.

### 2.3. Mitochondrial Dysfunction and Excitotoxicity

Mitochondrial failure induced by PM2.5 diminishes ATP synthesis and affects calcium equilibrium and redox signaling, intensifying hyperexcitability [75]. Furthermore, PM2.5 influences the expression and functionality of ionotropic glutamate receptors, particularly NMDA receptors, hence enhancing excitatory neurotransmission [76]. The activation of scavenger receptors in microglia exacerbates the internalization of PM2.5 and increases extracellular glutamate levels [76].

### 2.4. Interruption of GABAergic Signaling

A further essential process in epilepsy entails the interruption of γ‐aminobutyric acid [GABA] communication, the principal inhibitory neurotransmitter in the brain [77]. Research on animals indicates that exposure to PM2.5 results in the loss of GABAergic interneurons and a decrease in dendritic spine density in pyramidal neurons, hence compromising inhibitory tone and synaptic integration [78]. The resulting excitatory/inhibitory imbalance promotes epileptiform activity [79].

### 2.5. Hippocampal Injury and Oxidative Stress

Experimental models have demonstrated that PM2.5 causes anatomical and functional damage in the hippocampus, a critical region for seizure generation [80]. Elevated ROS in the hippocampus and cortex correlates with the onset and spread of seizure‐like discharges [81].

### 2.6. Signaling Pathways and Epigenetic Modifications

PM2.5 exposure disrupts significant signaling cascades, including the *Wnt*/β‐catenin and NF‐κB pathways [82, 83]. Dysregulation of the *Wnt*/β‐catenin pathway is associated with epileptogenesis, while NF‐κB modulates proinflammatory gene expression [83, 84]. PM2.5 stimulates MAPK family pathways JNK, ERK, and p38 MAPK through oxidative processes, leading to neuroinflammation and neuronal injury [85, 86]. These signaling disruptions also facilitate epigenetic modifications that may affect seizure susceptibility. Changes in DNA methylation and histone acetylation have been seen due to chronic PM2.5 exposure, specifically influencing genes such as TNF‐α, IL‐1, IL‐6, IL‐17, and IL‐10 [82, 83].

### 2.7. Novel Biomarkers

Novel biomarkers linked to PM2.5‐induced epileptogenesis encompass increased concentrations of proinflammatory cytokines (IL‐1β, IL‐6, TNF‐α), oxidative stress indicators (ROS, 4‐HNE), and modified expression of synaptic proteins, including NMDA receptor subunits and GABAergic markers such as GAD65 and GABRA1 [75, 80]. Structural indications, including hippocampus shrinkage and interneuron loss, have been identified as significant histological characteristics [87, 88].

### 2.8. Therapeutic Objectives

Potential therapeutic targets encompass antioxidant agents designed to restore mitochondrial function and redox equilibrium (e.g., Nrf2 activators), anti‐inflammatory medications that address microglial activation and NF‐κB signaling, and modulators of glutamatergic and GABAergic transmission to rectify excitatory/inhibitory imbalance [69, 79, 85]. Furthermore, epigenetic modulators, including histone deacetylase inhibitors, are under investigation for their potential to rectify PM2.5‐induced transcriptional dysregulation [89, 90].

## 3. AD

AD is characterized by the aggregation of β‐amyloid (Aβ) plaques and hyperphosphorylated tau neurofibrillary tangles, alongside mitochondrial dysfunction, neuroinflammation, and synaptic failure, resulting in gradual cognitive deterioration [91]. Exposure to PM2.5 has been associated with a heightened prevalence of AD, as these particles aggravate plaque accumulation and neurodegenerative mechanisms [92–94].

### 3.1. Synaptic Dysfunction and Amyloidogenic Pathway

Extended exposure to PM2.5 modifies neuronal structure and triggers synaptic reorganization, concurrently raising Aβ and tau biomarkers in cerebrospinal fluid and enhancing enzymes associated with the amyloidogenic pathway [36, 95, 96]. Oxidative stress, a key catalyst of Aβ oligomerization and tau phosphorylation, is exacerbated by PM2.5‐induced mitochondrial dysfunction [92].

### 3.2. Mitochondrial Dysfunction and Oxidative Stress

Compromised mitochondrial electron transport chain function elevates ROS levels, perpetuating a cycle of oxidative damage and neuronal apoptosis. This redox imbalance leads to neuronal death and intensifies the development of amyloid plaques [97, 98].

### 3.3. Disruption of PI3K/AKT Signaling

Moreover, the PI3K/AKT pathway, crucial for neuronal survival and synaptic integrity, is compromised, facilitating neurodegeneration. The suppression of this system diminishes neuronal resistance to oxidative stress and synaptic degradation [99].

### 3.4. Activation of the Hypothalamic–Pituitary–Adrenal (HPA) Axis in Response to Stress

The etiology of AD is exacerbated by glucocorticoid‐mediated stress responses triggered by PM2.5, which stimulate the HPA axis [100]. Increased glucocorticoids cause hippocampus shrinkage, cognitive deficits, and emotional disturbances [101]. Experimental evidence indicates spatial learning and memory impairments in mice subjected to PM2.5 exposure for 4 weeks [35].

### 3.5. Empirical and Transgenic Evidence

Chronic exposure to PM2.5 in transgenic AD mice models results in heightened Aβ deposition, tau phosphorylation, and microglial activation in memory‐associated areas such as the hippocampus and cortex [102].

### 3.6. Epigenetic Modifications and microRNA Dysregulation

Epigenetic modifications, including modified microRNA (miRNA) patterns, have also been implicated. Exposure to PM2.5 alters miRNAs such as miR‐338‐5p, let‐7e‐5p, miR‐99b‐5p, miR‐92b‐5p, and miR‐99a‐5p, which govern genes associated with synaptic development and cognitive function in fetal mice [103].

### 3.7. Activation of Microglia and Neurotoxicity

In vitro investigations of microglial cells reveal PM2.5‐induced neurotoxicity, characterized by increased ROS, apoptosis, and the production of inflammatory markers [60]. These results are corroborated by supplementary research demonstrating glial activation and Aβ buildup in mice exposed to PM2.5 [102, 104].

### 3.8. Clinical Evidence and Epidemiological Studies

Cohort studies, including senior populations, demonstrate that living in high‐PM2.5 regions correlates with an increased risk of AD, underscoring the necessity to consider air pollution as a modifiable risk factor for neurodegeneration [94, 105, 106]. Putative indicators associated with PM2.5‐induced AD pathology encompass elevated cerebrospinal fluid concentrations of Aβ42, phosphorylated tau, and proinflammatory cytokines (IL‐6, TNF‐α) [36, 37]. Mitochondrial dysfunction is indicated by reduced expression of respiratory complex subunits (e.g., COX‐IV), whereas oxidative stress biomarkers are characterized by elevated levels of 8‐OHdG and MDA [66]. Modified expression profiles of particular miRNAs (e.g., miR‐338‐5p, let‐7e‐5p) may serve as possible molecular biomarkers of early AD pathology [103].

### 3.9. Therapeutic Objectives

Possible therapeutic approaches encompass the administration of antioxidants (e.g., coenzyme Q10, N‐acetylcysteine) to mitigate mitochondrial ROS, anti‐inflammatory medicines aimed at activated microglia and NF‐κB signaling, and pharmacological compounds that modulate the PI3K/AKT pathway to reinstate synaptic resilience [60, 66, 99]. Epigenetic treatments, including miRNA mimics or inhibitors, are being explored to rectify transcriptome dysregulation caused by PM2.5 exposure [103, 107, 108].

## 4. PD

PD is a neurological condition marked by the gradual degeneration of dopaminergic neurons in the substantia nigra pars compacta and the buildup of Lewy bodies, predominantly consisting of α‐synuclein aggregates [109, 110]. The genesis of PD is multifaceted, involving both genetic and environmental variables, with exposure to PM2.5 identified as a key environmental risk factor linked to the disease’s development [31, 111–113].

### 4.1. Mitochondrial Dysfunction and Oxidative Stress in PD

Dopaminergic neurons in PD are especially susceptible to oxidative stress owing to their elevated metabolic activity, dopamine auto‐oxidation, and the presence of iron in the substantia nigra. This supplementary injury can hasten dopaminergic neuronal degeneration and intensify the motor impairments typical of PD [114, 115]. Recent findings indicate that heightened inflammation in the substantia nigra can expedite dopaminergic neuronal degeneration, exacerbating both motor and nonmotor symptoms of PD [116]. Furthermore, PM2.5 inhibits autophagy, an essential mechanism for the degradation of damaged proteins and defective organelles [115, 117]. Autophagy is impaired in PD due to mutations in genes such as LRRK2 and PINK1, together with the buildup of α‐synuclein [110, 118]. Mitochondrial dysfunction, a characteristic feature of PD, is another significant target of PM2.5. Exposure to PM2.5 has been documented to cause ultrastructural damage to mitochondria and myelin sheaths, elevate the expression of apoptosis‐related proteins such as caspase‐3 and caspase‐9, and disrupt neurobehavioral functioning [119]. These particles induce oxidative stress by disrupting the electron transport pathway, resulting in the overproduction of ROS [56]. Experimental models have shown that prolonged exposure to PM2.5 diminishes the function of essential mitochondrial complexes, particularly complex I, resembling the impairments seen in PD [120]. Moreover, PM2.5 can interfere with mitochondrial dynamics, encompassing fusion and fission processes vital for cellular stability. The disruption of these dynamics exacerbates neuronal dysfunction in PD, leading to increased dopaminergic neuronal death [121].

### 4.2. Aggregation of α‐Synuclein and Neurotoxicity

Prolonged exposure to PM2.5 in mice has been demonstrated to enhance dopaminergic neuronal degeneration in the substantia nigra, including increased levels of inflammatory and oxidative stress indicators [31]. Furthermore, human investigations have identified associations between extended PM2.5 exposure and a heightened chance of acquiring parkinsonian symptoms [112]. A study showed that PM2.5 can produce aggregated and hyperphosphorylated α‐synuclein in human olfactory neurons and critical brainstem nuclei [122]. PM2.5 may therefore facilitate modified α‐synuclein aggregation, resulting in neuronal impairment and the degeneration of dopaminergic neurons in the substantia nigra, which are possible toxicological pathways contributing to the initiation and progression of PD produced by PM2.5 [123].

### 4.3. Possible Biomarkers and Confounding Variables in PD

A cohort study in Ontario revealed that PM2.5 exposure (3.8 μg/m^3^) correlated with an elevated risk of PD [124]. Additional research has indicated elevated hospitalization rates attributable to PD. Research in New York indicates that prolonged exposure to PM2.5 can result in clinical worsening in PD, while the data imply that the detrimental effects of PM2.5 on PD may differ based on particle composition [112]. In PD, characterized by a genetic and epigenetic susceptibility to neuronal damage, PM2.5 may serve as a catalyst or aggravating element, hastening the start and advancement of symptoms. Age and inherent neurodegenerative susceptibility are substantial complicating variables that affect illness outcomes. Consequently, minimizing PM2.5 exposure, alongside the formulation of treatment techniques to alleviate its effects, such as focusing on mitochondrial function or α‐synuclein aggregation, may constitute a potential method for enhancing clinical results in PD patients [125, 126].

## 5. MS Disease

MS is an inflammatory and neurodegenerative disorder marked by demyelination, axonal degeneration, and gliosis [127]. The pathophysiology entails an immunological mechanism that attacks myelin and oligodendrocytes, resulting in gradual neuronal impairment [128]. The precise etiology of MS is multifaceted, involving both genetic and environmental influences; nonetheless, exposure to environmental pollutants such PM2.5 has been recognized as a potential environmental risk factor that aggravates disease progression [129, 130].

### 5.1. Immune Modulation and Oligodendrocyte Injury in MS

PM2.5 induces elevated ROS, compromising mitochondrial function and facilitating lipid peroxidation [131]. It diminishes the antioxidant capability of CNS cells, hence expediting axonal damage and demyelination [132]. In MS, oligodendrocytes and neurons experience considerable oxidative damage as a result of chronic inflammation [133]. The activation of peripheral and central nervous system immune cells, including autoreactive T lymphocytes, macrophages, and microglia, leads to the secretion of proinflammatory cytokines such as TNF‐α, IL‐1β, and IFN‐γ, which facilitate myelin degradation and oligodendrocyte apoptosis [134]. Moreover, these cytokines enhance inflammation by activating receptors including Toll‐like receptor 4 (TLR49 on microglia and astrocytes, thereby driving inflammatory cascades that exacerbate neuronal injury [135].

### 5.2. PM2.5 and Signaling Pathways in MS

The anomalous activation of NF‐κB by cytokines and oxidative stress facilitates persistent inflammation; PM2.5 exposure intensifies this signaling, worsening inflammatory pathophysiology [136]. Moreover, PM2.5 can influence the activity of the PI3K/AKT and mTOR pathways, impacting cell survival and autophagy, which are essential for the regulation of tissue repair in MS [99]. PM2.5 directly affects mitochondrial activity in central nervous system cells, exacerbating the energy and metabolic impairment linked to MS [32]. Experimental models indicate that PM2.5 disrupts mitochondrial dynamics, encompassing fusion and fission, and diminishes the activity of electron transport chain complexes, leading to excessive formation of ROS [43].

### 5.3. Epigenetic Modifications and Confounding Factors in MS

In MS, where mitochondria are already impaired by chronic inflammation and oxidative stress, changes produced by PM2.5 can expedite neuronal dysfunction and axonal degeneration [137, 138]. These epigenetic alterations may sustain persistent inflammatory conditions in MS, obstructing remyelination and exacerbating disease progression [139]. Certain studies indicate that PM2.5 exposure may modify the epigenetic control of essential genes in immunological and neural pathways, worsening demyelination and axonal injury [140, 141]. Clinical investigations have revealed an association between PM2.5 exposure and an elevated chance of acquiring MS or suffering exacerbated disease severity, particularly in urban regions with significant environmental pollution [129]. Confounding variables, including age, genetic predisposition, and concurrent autoimmune disorders, may affect these correlations.

PM2.5 constitutes a critical environmental variable affecting the advancement and intensity of MS [142]. These particles worsen the CNS’s susceptibility to the inflammatory and degenerative damage characteristic of MS through pathways including oxidative stress, neuroinflammation, mitochondrial dysfunction, and epigenetic changes [140]. Minimizing PM2.5 exposure, along with treatment interventions designed to alleviate its impacts, may constitute an advantageous strategy for enhancing clinical results in MS patients.

## 6. Huntington’s Disease (HD)

HD is an autosomal dominant neurological condition resulting from an aberrant amplification of CAG repeats in the HTT gene, which encodes the huntingtin protein. This condition is marked by progressive neuronal degeneration, especially in the striatum and cerebral cortex, resulting in motor, cognitive, and psychiatric deficits [143].

### 6.1. Inflammation and Excitotoxicity in HD

Exposure to PM2.5 may accelerate heart disease progression by elevating ROS, inducing neuroinflammation, and disrupting molecular processes in essential signaling pathways vital for neural homeostasis [1]. In HD, where neuronal susceptibility is already increased due to the toxic accumulation of mutant huntingtin (mHTT), PM2.5 intensifies neuronal injury via many inflammatory pathways [144]. Chronic inflammation is evident in HD patients as a result of microglial activation induced by mHTT [145]. Exposure to PM2.5 exacerbates the inflammatory response, deteriorating synaptic and neuronal function [136]. Recent findings indicate that heightened inflammation in the striatum, a critical area impacted in HD, expedites the degeneration of medium spiny neurons, which are especially susceptible in this condition [145].

### 6.2. Molecular Signaling and Potential Targets in HD

In HD, NF‐κB is dysregulated due to mHTT, fostering a proinflammatory and neurotoxic milieu [146]. PM2.5 also disturbs the equilibrium of neurotransmitters, including glutamate and GABA, hence influencing neuronal excitability [75, 147]. Despite being inadequately researched, existing studies indicate that PM2.5 may also affect genetic illnesses, including HD [142]. Research from animal models and initial human data indicate heightened cerebral injury and exacerbation of motor and cognitive symptoms [148]. Additional experimental and clinical investigations are necessary to comprehend this connection and its ramifications for HD patients.

## 7. Therapeutic Perspectives

Considering the extensive influence of PM2.5 on cerebral inflammation, particularly via the NLRP3 inflammasome pathway, therapeutics aimed at this mechanism may be beneficial. Blocking the NLRP3 pathway in AD may serve as a technique to mitigate the detrimental consequences of chronic pollution exposure. Investigating these biological targets may yield novel preventive or therapeutic strategies for many neurological disorders.

## 8. Emerging Biomarkers and Therapeutic Targets in PM2.5‐Related Neurological Disorders

**Figure 2:**
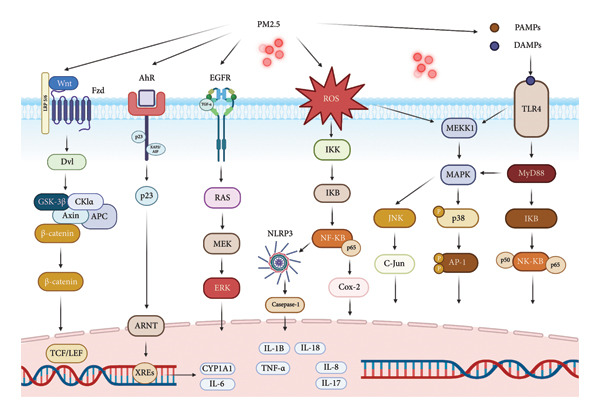
Signaling pathways involved by exposure to PM2.5. Exposure to PM2.5 leads to the dysregulation of multiple upstream and downstream cellular signaling pathways. Through PAMPs and DAMPs, TLR4 is activated, and together with ROS, they amplify the expression of MAPK and NF‐κB pathways upstream, generating inflammatory and apoptotic genes. AhR is stimulated by hydrocarbon‐derived particles, and through its chaperones, it translocates to the cell nucleus, forming a complex with ARNT. This complex binds to specific DNA sequences called xenobiotic response elements (XREs) in the promoter regions of target genes, leading to the expression of CYP1A and IL‐6. Finally, it increases the upstream expression of the Wnt/β‐catenin pathway, which has been found to be elevated in neurological diseases such as epilepsy. Created with https://www.biorender.com/.

Prolonged exposure to PM2.5 has been linked to several neuropathological pathways, many of which present potential opportunities for identifying specific biomarkers and treatment targets [142]. A burgeoning corpus of research indicates that PM2.5‐induced molecular and cellular modifications may function as both diagnostic markers and therapeutic targets in various neurological disorders [149] (Table 2). In epilepsy, increased concentrations of IL‐1β, IL‐6, TNF‐α, and oxidative stress indicators such as ROS and 4‐HNE are regarded as possible biomarkers of PM2.5‐induced neuroinflammation and mitochondrial dysfunction [48, 69, 153]. Moreover, the modified expression of synaptic proteins such as NMDA receptor subunits, GAD65, and GABRA1 indicates a disturbed excitatory/inhibitory equilibrium [75, 80]. Potential treatment targets encompass histone deacetylase inhibitors to rectify epigenetic dysregulation, alongside antioxidants and modulators of GABAergic and glutamatergic transmission [89, 90]. In AD, indicators including raised Aβ42 and phosphorylated tau levels in cerebrospinal fluid, together with COX‐IV downregulation and increased 8‐OHdG and MDA, signify mitochondrial and oxidative stress‐related damage [60, 66]. Dysregulated miRNAs [miR‐338‐5p, let‐7e‐5p] are becoming significant markers of PM2.5‐induced neurotoxicity [103]. Promising therapeutic techniques involve targeting the PI3K/AKT signaling pathway to improve neuronal survival and employing miRNA‐based therapeutics to restore gene expression equilibrium [99]. In PD, indicators including α‐synuclein buildup, complex I inhibition, and increased caspase‐3/9 levels signify PM2.5‐associated neurodegeneration [117, 119, 123]. Impaired mitochondrial dynamics [imbalance between fusion and fission] and disrupted autophagic flow have also been emphasized [43, 121]. Therapeutically, agents that restore mitochondrial function (e.g., CoQ10, MitoQ), enhance autophagy, or decrease proinflammatory mediators such as NF‐κB may prove advantageous [154]. In MS, although not thoroughly addressed in the present article, preliminary findings suggest that PM2.5 may aggravate demyelination and T‐cell‐driven immunological responses, with biomarkers such as elevated IFN‐γ, IL‐17, and CXCL10 being pertinent [155]. Possible therapy strategies may include antioxidants, immunological modulators, and Nrf2 activators to address redox imbalance and neuroinflammation [156] (Table 3). Identifying and targeting disease‐specific molecular markers enable the development of more precise diagnostic tools and individualized therapy techniques to mitigate the neurological effects of PM2.5 exposure (Figure 2). This focused strategy represents a considerable improvement over conventional toxicological evaluations and facilitates the evolution of environment‐based precision medicine.

**Table 2:** Critical experimental parameters extracted from key studies on PM2.5 exposure and its effects on neurological disorders.

Study	Model	PM2.5 source	Concentration/exposure	Exposure duration	Endpoints evaluated/main findings
[150]	Mice	Urban PM2.5	300 μg/m^3^	6 months (chronic)	Neuroinflammation, cognitive function
					↑ IL‐1β, TNF‐α; memory impairment

[41]	Mice	Traffic‐derived PM2.5	100 μg/m^3^	10 weeks	Learning and memory, hippocampal morphology
					↓ hippocampal spine density, impaired cognition

[36]	Humans (autopsy)	Urban air	NA	Chronic (lifetime exposure)	Neuropathology, β‐amyloid, tau
					Early Alzheimer‐like pathology in exposed children

[151]	Rats	Standardized PM2.5	5 mg/kg	4 weeks	Oxidative stress, mitochondrial function
					↑ ROS, ↓ mitochondrial membrane potential

[152]	Humans	Urban air	NA	Long‐term exposure	Magnetite nanoparticles in brain
					PM2.5‐related iron‐rich particles in frontal cortex

*Note:* Essential experimental parameters derived from significant research assessing the impact of PM2.5 exposure on neurological diseases. The table encapsulates animal and human studies, outlining the experimental models, sources of PM2.5, exposure concentrations and durations, assessed endpoints, and principal findings. The data demonstrate a consistent association between PM2.5 exposure and neuroinflammatory processes, cognitive deficits, oxidative stress, and early neuropathological alterations related to neurodegenerative disorders.

**Table 3:** Inconsistencies in PM2.5‐related studies based on concentration, model used, and molecular outcomes.

Study	Model	PM2.5 concentration	Exposure type/duration	Molecular outcome	Reported effect
[151]	Rats	5 mg/kg	4 weeks (acute/subchronic)	Oxidative stress	↑ ROS, ↓ mitochondrial function
[157]	Mice	4 mg/mL	2 weeks	Nrf2 pathway activation	↑ Nrf2, ↑ HO‐1 expression
[158]	Mice	100 μg/m^3^	3 months	Apoptosis markers	↑ caspase‐3, ↑ Bax
[159]	Mice	500 μg/m^3^	12 months	Antioxidant enzymes	↑ SOD, ↑ catalase (adaptive response)
[160]	Rats	1 ppm	5 weeks	Neuroinflammation	No significant change in IL‐1β or TNF‐α

*Note:* Discrepancies in PM2.5‐related research concerning concentration, employed models, and molecular outcomes. The table underscores the heterogeneity in experimental results across several research projects, maybe attributable to discrepancies in exposure dose, duration, animal models, or the sensitivity of the examined biochemical pathways. These discrepancies underscore the necessity for standardization in experimental design to enhance comparability and reproducibility.

## 9. Conclusion

Acceptable PM2.5 particles represent a significant risk to cerebral health owing to their capacity to traverse biological barriers and infiltrate the central nervous system. Upon entry, they initiate a series of deleterious processes, encompassing oxidative stress, chronic inflammation, mitochondrial malfunction, and alterations in gene regulation and cellular signaling. Experimental and clinical investigations have associated continuous PM2.5 exposure with an elevated risk and accelerated progression of neurological disorders, including AD, PD, HD, MS, and epilepsy. These findings underscore the pressing necessity for more stringent environmental restrictions to mitigate PM2.5 exposure. Simultaneously, additional study is required to ascertain viable therapeutics that mitigate these consequences, particularly those aimed at inflammation, oxidative stress, and epigenetic disturbance.

## Ethics Statement

According to international standards, written ethical approval has been collected and preserved by the author(s).

## Consent

The authors have nothing to report.

## Conflicts of Interest

The authors declare no conflicts of interest.

## Author Contributions

Carmen Rubio: original draft preparation, conceptualization, and review and editing.

Alejandro López‐Landa: methodology and figures.

Norma Serrano‐García: review and editing.

Héctor Romo‐Parra: review and editing.

Moisés Rubio‐Osornio: original draft preparation, conceptualization, and review and editing.

## Funding

The authors received no specific funding for this work.

## Data Availability

Data sharing is not applicable to this article as no datasets were generated or analyzed during the current study.
